# Postoperative COVID-19 Pneumonia following Resection of a Large Thoracic Chondrosarcoma

**DOI:** 10.1155/2021/8866848

**Published:** 2021-01-23

**Authors:** Austin C. Kaidi, Michael B. Held, Paul J. Park, Wakenda K. Tyler

**Affiliations:** Department of Orthopedic Surgery, Columbia University Medical Center, 622 W. 168th St, PH-11, New York, NY 10032, USA

## Abstract

**Case:**

A 57-year-old man presenting with two months of insidious shoulder pain was found to have a large thoracic chondrosarcoma invading the spinal canal. The patient's orthopedic oncologist organized an interdisciplinary team including interventional radiology, thoracic surgery, neurosurgery, and plastic surgery. This allowed safe, en bloc tumor resection. The patient's postoperative course was complicated by COVID-19 pneumonia, which was rapidly identified and medically managed with full recovery.

**Conclusion:**

Postoperative COVID-19 pneumonia can present insidiously and mimic other postoperative complications. Early identification and testing can promote rapid isolation, proper personal protective equipment use, and guide outcome-improving treatments.

## 1. Introduction

The COVID-19 pandemic has called into question how orthopedic surgeons should safely practice nonemergent surgeries. To date, there are few documented cases of postoperative COVID-19 infection following orthopedic procedures [1]. In this case, we present a thoracic chondrosarcoma complicated by postoperative COVID-19 pneumonia.

Chondrosarcomas are a group of malignant neoplasms comprised of tumor cells that produce cartilaginous matrix. The only proven treatment is definitive surgical resection [2]. Primary chondrosarcomas make up about 75% of all chondrosarcomas, but they can also be seen in the setting of multiple enchondromatoses, such as Ollier disease or Maffucci syndrome [3]. Chest wall chondrosarcoma excisions require complex surgical approaches to avoid damaging critical nearby structures. During the COVID-19 pandemic, postoperative recovery in a surgical intensive care unit (SICU) can increase a patients' risk of COVID-19 exposure from providers and other patients [4, 5]. In this case, we highlight the need for swift recognition and coordinated management of postoperative COVID-19 infections to allow a positive outcome.

The patient was informed that his case would be submitted for publication and provided consent.

## 2. Case History

**Figure 1:**
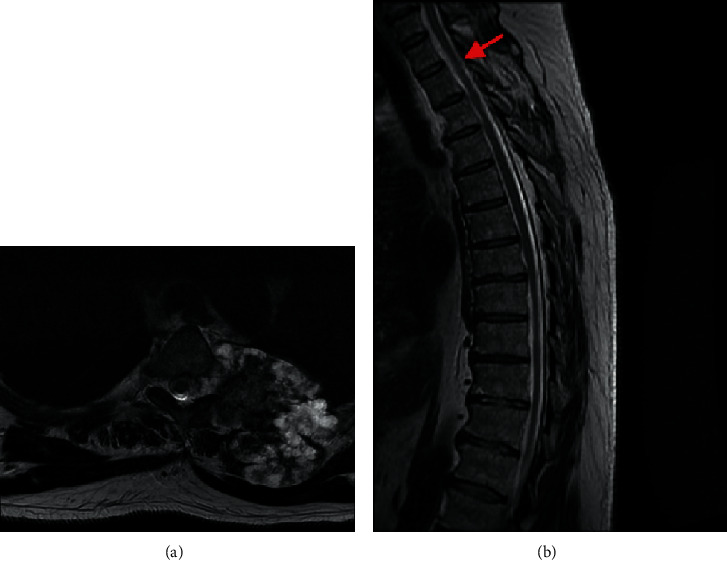
T2 MRI of patient's thoracic spine: (a) axial view at T4 level; (b) midline sagittal with arrow at T4.

A 57-year-old male with hypertension presented to a nonoperative physician complaining of two months of intermittent left shoulder pain localized to the scapula and lateral shoulder. He reported no weakness or numbness upon initial evaluation, and the working diagnosis was scapular dyskinesis. The patient initially underwent one month of physical therapy and nonsteroidal anti-inflammatory drugs (NSAIDs) without improvement. Subsequent computed tomography (CT) scan and magnetic resonance imagining (MRI) demonstrated a large exophytic mass originating at T3 with extension cranially to C5, caudally to T5-T6, and intrathoracic extension. The mass measured approximately 6.7 cm × 4.4 cm × 7.7 cm and extended into the spinal canal with abutment of the spinal cord at T3-T4 (Figure 1). No metastatic chest disease was appreciated on either study. The patient was referred to an orthopedic oncologist for further evaluation. Interval history at this time demonstrated radiating numbness and discomfort in the left axilla. The patient's orthopedic oncologist organized an interdisciplinary surgical care team for safe resection.

### 2.1. Preoperative Planning

The size, location, and proximity of tumor to vulnerable anatomical structures warranted a multidisciplinary, coordinated approach. This care team was organized by the patient's orthopedic oncologist and included interventional radiology (IR), neurosurgery, thoracic surgery, plastic surgery, and internal medicine.

**Figure 2:**
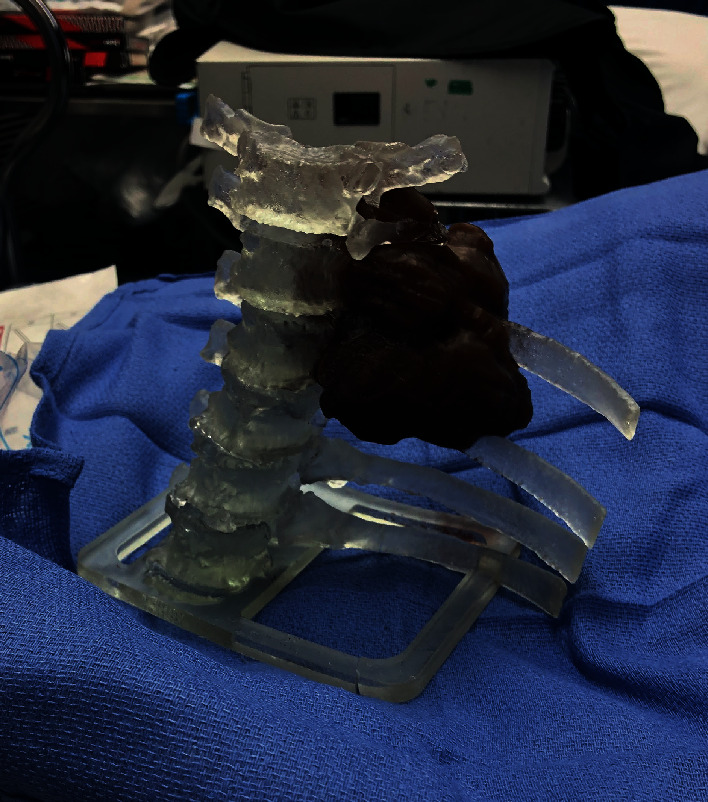
3-D model of patient's thorax.

First, IR conducted a CT-guided biopsy of the mass. Pathology demonstrated lobulated hyaline cartilage suspicious for low-grade chondrosarcoma. IR also placed an inferior vena cava (IVC) filter to prevent embolic sequelae. The patient was seen preoperatively by thoracic surgery and neurosurgery for multidisciplinary surgical planning. In order to successfully conduct an en bloc resection, it was decided that the operation would need to be sequentially staged using a lateral and posterior approach. The lateral stage was performed first in conjunction with thoracic surgery, the posterior stage was performed next with neurosurgery, and closure was done with plastic surgery. A 3-D printed model of the patient's thorax was generated to assist in preoperative planning (Figure 2).

### 2.2. Stage 1: Lateral Resection

On the morning of surgery, the patient was afebrile without respiratory complaints, and there was no government or hospital mandate to postpone elective cases. Mandatory preoperative COVID-19 testing was also not standard protocol, as this was prior to widespread outbreak. The patient was positioned right-lateral decubitus, and all teams including orthopedic oncology, thoracic surgery, neurosurgery, and plastic surgery were present for planning and marking the incisions. This was critical to ensure the thoracotomy would not interfere with the spinal incision and that an adequate skin bridge was left to preserve coverage options for plastic surgery.

Thoracic surgery initially evaluated for intra- and extrapulmonary metastases utilizing flexible bronchoscopy and a video-assisted thoracoscopic surgery (VATS) system. Both were negative. VATS was used to identify ribs 3-5 for resection. Thoracotomy was performed after discussion with orthopedics, neurosurgery, and thoracic surgery. Osteotomies were performed along the lateral aspects of vertebral bodies T2-T4 using an ultrasonic bone scalpel with a margin. Due to the tumor's position, surgeons were unable to make a complete cut through the vertebral body. The bone cuts were taken as far as possible medially and posteriorly using the bone scalpel and curved osteotomes. At this point, the tumor was free anteriorly, a chest tube was inserted, and the thoracic wound was closed.

### 2.3. Operative Stage 2: Posterior Resection

**Figure 3:**
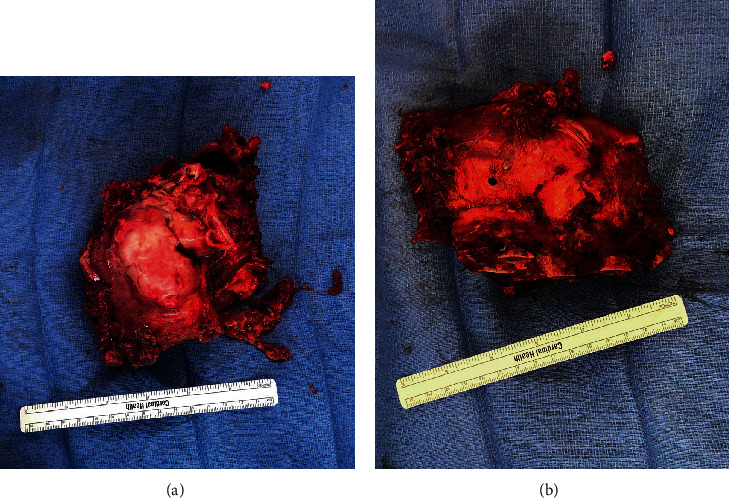
Removed tumor: (a) anterior surface; (b) posterior surface.

**Figure 4:**
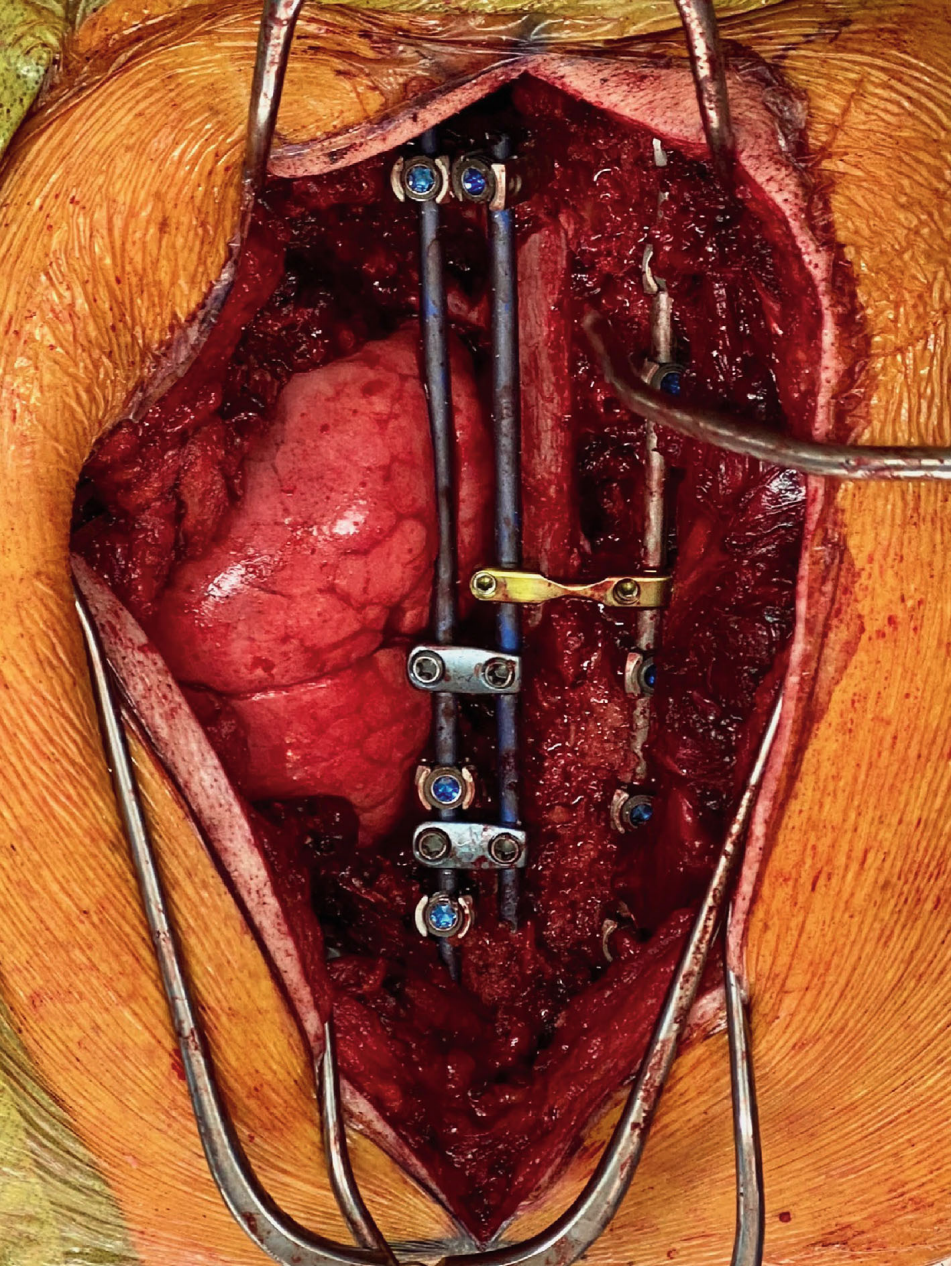
Image of posterior midline incision after removal of tumor and spinal fusion.

For stage 2, the patient was positioned prone on a Jackson table with neuromonitoring. A posterior midline incision was made as previously marked at the case's start. Posterior laminectomies were performed from the inferior portion of C7 to T4. The T2-T4 nerve roots were divided, the spinal cord was mobilized to the right, and transpedicular decompression was performed from T1 to T4. Posterior osteotomies heading anteriorly were connected with the osteotomies made in the lateral position. This allowed for a complete en bloc resection of the tumor (Figure 3). Posterolateral spinal fusion was done from C7 to T6 (Figure 4). Hydrogen peroxide irrigation was performed in case of local contamination, and the tumor was sent for pathological evaluation demonstrating a grade 1, stage pT1, and pN0 chondrosarcoma with negative bone and soft tissue margins.

For closure, plastic surgery had initially considered a latissimus dorsi flap. Given the careful planning at the case's onset however, it was possible to perform a paraspinal muscle advancement flap with local tissue rearrangement to avoid the more time-intensive, debilitating rotational flap.

### 2.4. Postoperative Course

**Figure 5:**
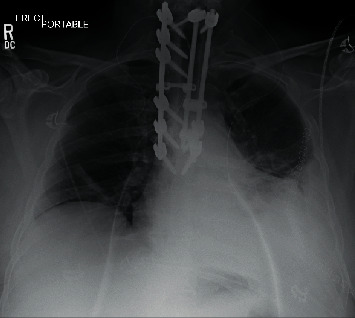
Chest X-ray of patient with COVID-19 pneumonia.

Operative duration was greater than 16 hours, and estimated blood loss was 3.3 L. The patient was taken to the SICU postoperatively. He was neurologically intact, except for expected chest wall numbness. The patient was extubated on postoperative day (POD) 2 and transferred to surgical step-down (SSD) on POD4 for close monitoring. Chest tubes were removed on POD5, with follow-up chest X-ray (CXR) demonstrating no pneumothorax or effusion. The postoperative course was unremarkable until POD7, when he was febrile to 39.4°C with a new oxygen requirement (3 L nasal canula (NC) from previous room air). CXR on POD7 also identified a new, left-sided effusion with diffuse bilateral infiltrates (Figure 5).

On POD7, COVID-19 prevalence was low in New York State, with fewer than 4,000 confirmed cases. However, the patient's fever and oxygen requirement raised suspicion. Other considered diagnoses included surgical site infection, bacterial pneumonia, and pulmonary embolism. On POD7, a COVID-19 nasal swab was obtained, and it returned positive for COVID-19. Medical teams were immediately asked to help comanage. Thoracentesis was deemed unnecessary in the setting of a positive COVID-19 diagnosis.

The COVID-dedicated infectious disease (ID) team had been utilizing tests like ferritin, CRP, and D-dimer to assess infection severity and guide treatment. In a postoperative patient with malignancy, it was difficult to interpret these elevated, nonspecific values. Although the patient had mild symptoms, the COVID-dedicated team recommended treatment with hydroxychloroquine 600 mg twice daily for 3 days (then 400 mg for 4 days) and azithromycin 250 mg daily for 5 days, after ruling out baseline QT-prolongation on EKG. This decision was determined by the patient's risk factors for decompensation, including intrathoracic surgery and malignancy history.

The patient remained febrile through POD10 and had increasing oxygen requirements to 6 L NC. Upon requiring 6 L, he was transferred to the medicine service with surgical comanagement. To minimize provider exposure, physical therapy (PT) was halted and only one surgical team evaluated the patient daily. By POD19, he had completed treatment, remained afebrile, and was breathing on room air. He restarted PT, began ambulating, and was transferred to acute inpatient rehabilitation. 12-week postoperatively, the patient is progressing well at home without further pulmonary complications.

## 3. Discussion

Chondrosarcomas make up approximately 20-27% of all primary malignant bone tumors [2, 6]. Even among rare chest wall chondrosarcomas, costochondral tumors are more common than vertebral or costotransverse tumors. Only select case reports have documented chondrosarcomas similar to the one seen in this report [3, 7–10]. This case is unique for its location, the multidisciplinary surgical approach utilized, and the development of postoperative COVID-19 pneumonia. We believe this case provides insight regarding postoperative management of orthopedic patients during the COVID-19 pandemic and the importance of an orthopedic oncologist “quarterbacking” a patient's care. Outcomes can be improved with a dedicated COVID ID team. Shorter ICU and SSD stays may also lower nosocomial infection risk, particularly when hospitals are inundated with COVID-19 patients.

Currently, the literature on postoperative COVID-19 infections following orthopedic procedures is limited. Rabie et al. reported 7 orthopedic trauma patients who tested COVID positive on presentation. Two of the patients expired; however, both had nonoperatively treated hip fractures [1]. This study is limited as it addresses neither postoperative infections nor treatment. Mi et al. studied 10 orthopedic fracture patients who tested COVID positive preoperatively and evaluated whether surgical intervention was safe [11]. In this study, 3 patients expired, two of which were also nonoperatively treated hip fractures. This study did not evaluate postoperative infections. Chang et al. evaluated surgical interventions on fracture patients in Singapore [12]. They also did not address postoperative infections but did recommend utilizing inflammatory lab markers in COVID-positive patients to determine a patient's surgical risk.

In this report, our patient developed symptoms on POD7, indicating a nosocomial or preoperative infection, given known 14-day incubation periods [13]. This emphasizes the importance of JAAOS guidelines regarding mandatory preoperative COVID-19 testing for nonemergent cases. Had this patient tested positive preoperatively, the case would have been postponed until infection resolution [14].

Given the patient's risk for serious complications in the setting of malignancy and major surgery, the team had a low threshold for COVID testing. This patient was diagnosed with COVID-19 on the same day of symptom presentation, even though the initial differential diagnosis was broad given his nonspecific symptoms of a mild oxygen requirement, fever, and pleural effusion. A low threshold for testing allowed rapid COVID team consultation and patient isolation. Although hydroxychloroquine and azithromycin have shown to be ineffective treatments, we believe dedicated COVID team comanagement improved this patient's outcome [15]. Decisions to escalate care were difficult in this patient, since standard COVID-19 laboratory values were unreliable due to his malignancy and postoperative inflammation. A dedicated COVID team was able to more closely assess the patient, monitor for decompensation, and rapidly escalate ventilatory support when needed.

Due to his COVID-19 pneumonia, this patient's overall postoperative timeline was drastically prolonged, and he remained largely bedbound for three weeks. This made preoperative IVC filter placement advantageous for reducing the risk of embolic complication [16, 17]. At our institution, IVC filters are routinely placed for large pelvic, sacral, and spinal sarcoma cases, since postoperative anticoagulation often must be held. If put on anticoagulation, patients' large surgical spaces can form diffuse hematomas requiring multiple transfusions. Our recommended indications for preoperative IVC filter placement are as follows: (1) operations expected to be longer than 8 hours; (2) operations expected to require 4 or more units of blood products; (3) operations expected to require prolonged postoperative ICU stays; or (4) operations requiring spinal instrumentation, where postoperative epidural bleeding could cause neural tissue compromise.

Finally, this case highlights the need for an orthopedic oncologist “quarterbacking” care. The orthopedic oncologist coordinated care from preoperative planning to postoperative management and was scrubbed for the entirety of the procedure. This allowed for preoperative placement of an IVC filter and intraoperative closure with an advancement flap, sparing the patient a rotational latissimus flap, which carries the risk of flap failure [18]. Postoperatively, the decision to COVID-test early on allowed for coordination of care with a dedicated COVID team who had more expertise in treating critically ill patients.

This report describes a unique primary chondrosarcoma and provides insight on the management of postoperative COVID-19 pneumonia in orthopedic surgery. We recommend universal preoperative testing, short ICU stays when possible, and comanagement with a dedicated COVID team for COVID-positive patients. This can optimize provider protection and patient outcomes.

## Data Availability

The paper describes a case report of a patient that was treated by the authors.
